# Molecular characterization and improved diagnostics of *Nocardia* strains isolated over the last two decades at a German tertiary care center

**DOI:** 10.17179/excli2021-3787

**Published:** 2021-04-30

**Authors:** Patrick Chhatwal, Sabrina Woltemate, Stefan Ziesing, Tobias Welte, Dirk Schlüter, Marius Vital

**Affiliations:** 1 Institute for Medical Microbiology and Hospital Epidemiology, Hannover Medical School (MHH), Hannover, Germany; 2 Department of Pneumology and German Center for Lung Research, Hannover Medical School (MHH), Hannover, Germany

**Keywords:** Nocardia, whole genome sequencing, resistance mechanisms, molecular diagnostics

## Abstract

Nocardiosis is a rare but life-threatening infection caused by aerobic *Actinomycetes *of the genus *Nocardia* particularly affecting immunocompromised hosts. The identification of *Nocardia ssp.* and antibiotic susceptibility testing by standard microbiological methods are incomplete and molecular techniques may improve diagnostics. We studied 39 *Nocardia* strains isolated from 33 patients between 2000 and 2018. Twenty-four patients (72.7 %) were immunocompromised. Whole genome sequencing (WGS) revealed a broad taxonomic range of those isolates spanning 13 different species, including four strains that belonged to three novel species based on average nucleotide identity (ANI < 95 % with currently available genome sequences). 16S rRNA gene analyses mirrored WGS results. Conventional MALDI-TOF analysis correctly identified 29 isolates at the species level (74.4 %). Our advanced protocol with formic acid and acetonitrile treatment increased identification to 35 isolates (89.7 %). Antibiotic resistance was tested using both a microdilution method and MIC strip testing. Results were in good concordance with an overall trimethoprim-sulfamethoxazole (SXT) resistance rate of 13.5 %*.* WGS of a SXT resistant *N. farcinica* isolate showed a deletion of several amino acids in a homolog of dihydropteroate synthase (FolP2) that was not seen in sensitive members of this species. Diversity of *Nocardia* isolates was high and involved many different species, suggesting that this taxon has broadly distributed mechanisms for infecting individuals. Widely applicable diagnostic methods including MALDI-TOF and 16S rRNA gene analyses correctly identified most strains. WGS additionally revealed molecular insights into SXT resistance mechanisms of clinical *Nocardia* isolates highlighting the potential application of (meta)genomic-based diagnostics in the future.

## List of abbreviations

AMK - Amikacin

ANI - Average nucleotide identity

AST - Antibiotic susceptibility testing

CLSI - Clinical & Laboratory Standards Institute

LZD - Linezolid

MIC - Minimal inhibitory concentration

MXF - Moxifloxacin

SXT - Trimethoprim-sulfamethoxazole

WGS - Whole genome sequencing

## Introduction

*Nocardia* are gram-positive, partially acid-fast aerobic rods belonging to the order of *Actinomycetales*. They occur ubiquitously in soil and in wet biotopes and are considered as opportunistic pathogens that cause life-threatening infections, particularly in immunocompromised hosts (Mehta and Shamoo, 2020[18]). In recent studies, an increase of nocardiosis in non-immunocompromised hosts, especially bronchiectasis patients, has been described (Ambrosioni et al., 2010[2]; Woodworth et al., 2017[29]; Restrepo and Clark, 2019[24]). Inhalation of *Nocardia* is considered as the main route of infection. Therefore, nocardiosis primarily manifests in the lung, but extrapulmonary dissemination into other organ systems (e.g. the central nervous system) is possible as well. 

Microbiological detection of *Nocardia* is challenging, because *Nocardia spp.* from respiratory specimens often grow very slowly and require selective culture media. Due to their slow growth, it usually lasts several days until *Nocardia* are identified, and up to five additional days for antibiotic susceptibility testing to be finished (Du et al., 2016[10]). In the recent past, several methods for identification of cultivated *Nocardia* have been proposed, including molecular methods such as Multilocus Sequence Typing (MLST) (Baio et al., 2013[3]; Du et al., 2016[10]) and 16S rRNA gene sequencing (Helal et al., 2011[13]), as well as mass spectrometry-based methods (MALDI-TOF) (Girard et al., 2017[12]; Marín et al., 2018[15]) MALDI-TOF is a rapid and cost-efficient method for identification of cultured bacteria; however, the utility heavily relies on the availability of well-developed and validated databases (Body et al., 2018[6]). Therapy of nocardiosis is based on long-term antimicrobial treatment using trimethoprim-sulfamethoxazole (SXT) or combinations of other antibiotics for 6 - 12 months (Restrepo and Clark, 2019[24]). Due to increasing resistance rates against SXT (Uhde et al., 2010[27]) treatment becomes increasingly challenging.

In the present study, we characterized *Nocardia* strains isolated from 2000-2018 in a tertiary care university hospital based on whole genome sequencing (WGS). Additionally, applicability of routinely available techniques, including 16S rRNA gene analysis and an improved MALDI-TOF protocol, for the identification of *Nocardia* species and the determination of antibiotic susceptibility, in particular for SXT resistance, was assessed. Results were compared with traditional culture-based methods. 

## Materials and Methods

### Collection of Nocardia strains

The Hannover Medical School - a 1,500-bed university hospital in northern Germany is a large transplantation center with > 400 solid organ transplantations (> 140 lung transplantations) per year. We included *Nocardia spp.* strains from patient specimens that were collected between the years 2000 and 2018. Orthologous strains, which were cultured from the same patient within one month, were excluded from further analysis. Strains collected from patients without informed consent were also excluded (n = 11). One further strain could not be re-cultured after freezing. The study was approved by the local ethics committee (No. 8504_BO_K_2019). 

### DNA Extraction and whole genome sequencing

Eight *Nocardia* strains (P01_1, P02_1, P03_1, P19_1, P21_1, P22_1, P23_1, and P32_5) were cultured in Brain-Heart-Infusion (BHI) supplemented with Vitox (Oxoid, Wesel, Germany). The remaining strains were cultured in LB-broth (Lennox; Becton Dickinson, Heidelberg, Germany) with 0.05 % Tween20 (AppliChem, Darmstadt, Germany). One ml from the overnight cultures was transferred to 20 ml BHI or LB/Tween20 and incubated for 1-3 days. Two ml of this culture was centrifuged, washed twice with NaCl (0.9 %), resuspended in 500 µl NaCl (0.9 %) and stored at -20 °C until DNA preparation. DNA preparation was performed with 0.1 mm silica beads and with phenole-chloroform-isoamylalcohol (24:25:1) extraction. Library preparation was done with Nextera XT DNA preparation kit (Illumina, San Diego, USA) according to manufacturer's manual with slight modifications: DNA input was increased to 1.5 ng, tagmentation time was extended to 5 min 35 sec and final elution volume was decreased to 35 µl. Quality control was performed with D5000HS (HighSensitivity) screen tapes on the 4200 TapeStation system (Agilent, Santa Clara, USA). DNA concentrations were determined with the Qubit dsDNA HighSensitivity assay (Thermo Fisher Scientific, Dreieich, Germany). Libraries were diluted to 4 nM and 5 µl of each strain were pooled. Libraries were pooled equimolar to three pools according to the expected genome size (< 7 Mbp, 7-8.5 Mbp, > 9.5 Mbp) and sequenced with an approximated coverage of 120 on an Illumina Sequencer (MiSeq v3). 

### Bioinformatic processing

Raw reads were quality filtered via fastp (Chen et al., 2018[7]), assembled using SPAdes (Bankevich et al., 2012[4]) and quality checked (completeness/contamination) using CheckM (Parks et al., 2015[21]). Subsequent gene calling was performed with prokka (Seemann, 2014[26]) and the Average Nucleotide Identity (ANI) including all available *Nocardia* reference genomes (n = 157) at the Genome Taxonomy Database (GTDB) was calculated using fastANI (Jain et al., 2018)[14]. A dendrogram including all isolates, all closest matching references, as well as all representative genomes of individual *Nocardia* species was calculated in R with ggtree (Yu et al., 2017[30]). Two reference strains purchased from the DSMZ (German Collection of Microorganisms, Braunschweig, Germany), *N. lasii* DSM 100525 and *N. wallacei *DSM 45136, were included as well. The 16S rRNA gene sequences of all isolates and references were deduced from prokka results and a phylogenetic tree including the same genomes from above was calculated with fasttree (Price et al., 2010[23]).

### MALDI-TOF identification

*Nocardia* strains were incubated for 24-72 hours on Columbia blood agar (Becton Dickinson, Heidelberg, Germany) until colonies were visible. For MALDI-TOF-based identification, we used two different protocols. (I) A bacterial colony was spotted onto a polished steel MALDI-TOF target plate. Samples were overlaid with 0.5 µl of formic acid (BioMérieux, Nuertingen, Germany) and, once dried, 1 µl of matrix (CHCA, alpha-cyano-4-hydroxy-cinnamic acid) was added. (II) A bacterial colony was suspended in 10 μl of 70 % formic acid and subsequently in 10 μl of pure acetonitrile. After a centrifugation step of 2 minutes at 14,000 x g, 1 µl of the supernatant was spotted onto the MALDI-TOF target plate. Once dried, 1 µl of matrix (CHCA, alpha-cyano-4-hydroxy-cinnamic acid) was added. All isolates were analyzed in duplicates. 

### Antibiotic susceptibility testing

For culture-based antibiotic susceptibility testing, we compared two methods. For both methods we used *N. nova* ATCC BAA-2227 as control strain. *Nocardia* strains were incubated for 24-72 hours on Columbia blood agar (Becton Dickinson, Heidelberg, Germany) until growth was visible. (I) According to the CLSI M62 performance standards of susceptibility testing, we performed a microdilution method using Sensititre™ Myco RAPMYCO AST-Plate (Thermo Fisher Scientific). According to the manufacturer's instructions, *Nocardia* were suspended in demineralized water (McFarland 0.5). Fifty µl of the suspension was transferred to cation adjusted Mueller-Hinton broth with TES (Thermo Fisher Scientific). One hundred µl of the suspension was transferred to each well of the microtiter plate. MICs were determined visually after 48 h-72 h until bacterial growth was visible. (II) *Nocardia *were suspended in demineralized water (McFarland 0.5). A sterile cotton swab was dipped into the inoculum suspension and the inoculum was spread over the entire surface of the Mueller-Hinton agar plate (Oxoid) by swabbing in three directions. MIC Test strips (bestbion dx, Cologne, Germany) were applied firmly on the plate. MICs were determined visually after 48 h-72 h when bacterial growth was visible.

Very major errors were defined as observed resistance via microdilution test where MIC test stripe revealed a sensitive result. Major errors were defined as sensitive microdilution test result whereas MIC test stripe revealed a resistant result. Minor errors were defined as discrepancies of more than one titration step between both methods, but both methods showed the same categorical result (resistant / intermediate / susceptible).

## Results

### Baseline characteristics of nocardiosis patients

33 nocardiosis patients were included in this study (Table 1(Tab. 1)). The median age was 46 years and 25 patients (76 %) were immunocompromised. The main reason for immunosuppression was lung transplantation (16 patients, 49 %). Six patients (18 %) had other solid organ transplantations and two patients (6 %) had a hematologic stem cell transplantation. The median time interval between transplantation and diagnosis of nocardiosis was 13.5 months (range 1-121 months). Twenty-two patients (67 %) had a chronic lung disease, of these five patients (15 %) had cystic fibrosis and two patients (6 %) had bronchiectasis. Three patients (9 %) did not have any known risk factors for nocardiosis. We did not see an increasing or decreasing incidence over time and cultured isolated 1-8 *Nocardia* strains from patient specimens per year. Twenty-five patients (76 %) had a pulmonary nocardiosis. Two of these patients (6 %; P19 and P26) remained culturally positive in follow-up samples that were taken three months after the first positive culture, and one lung transplanted patient (P32) remained positive for five years. 

### Phylogeny of Nocardia isolates and reference strains

**Figure 1:**
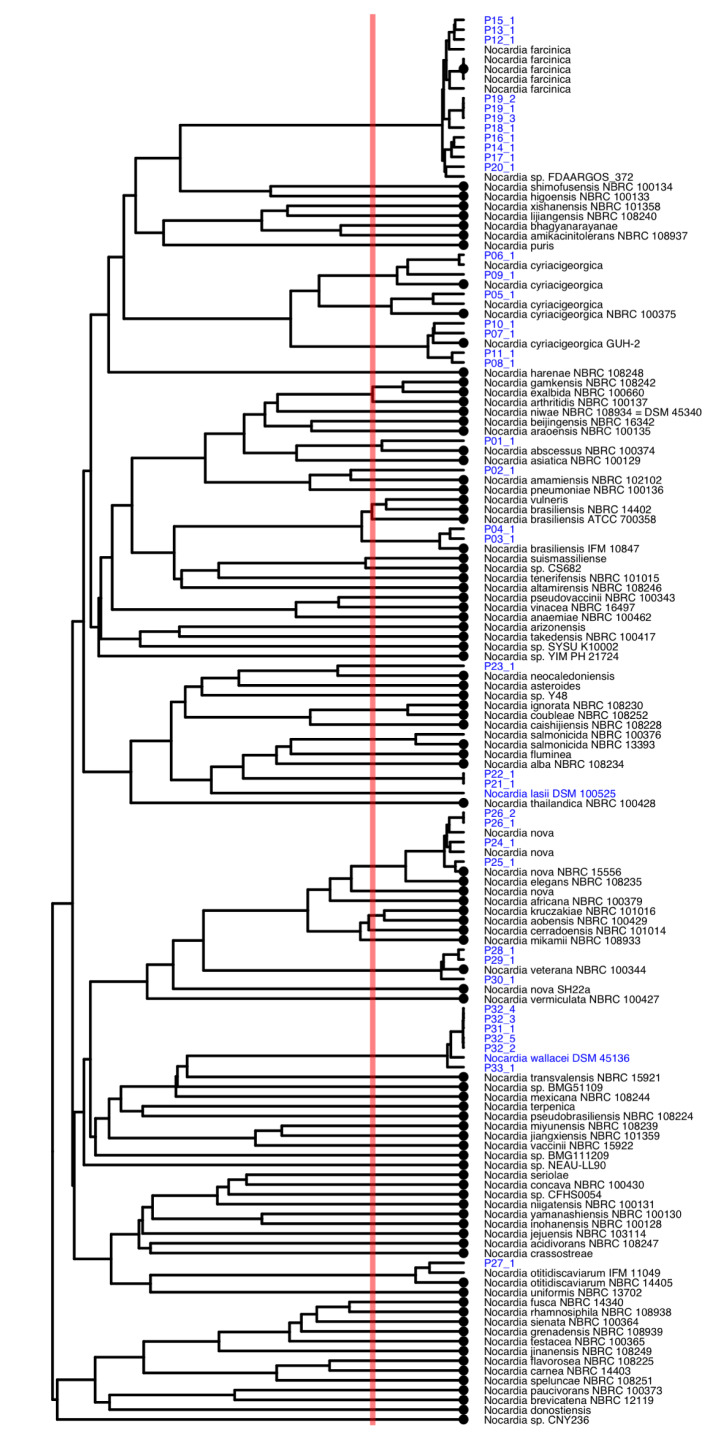
Dendrogram based on Average Nucleotide Identities (ANI) between all isolates of this study (blue) and *Nocardia* reference genomes of the Genome Taxonomy Database (GTDB), where all closest matching references as well as all representative genomes of individual species (highlighted with a dot at the tip) were included. The two references from the German Collection of Microorganisms (DSMZ) sequenced in this study are shown in blue. The red line depicts the ANI cut-off value of 95 % that is suggested to represent the species cut-off.

Figure 1(Fig. 1) gives an overview of phylogenetic relation of *Nocardia* strains isolated in this study along publicly available *Nocardia* genomes. In total, the Genome Taxonomy Database (GTDB), which is an effort to provide up-to-date taxonomy of bacteria based on their phylogenetic relations on the genome level, counts 157 *Nocardia* genomes that are clustered into 92 species (Parks et al., 2020[20]). Based on GTDB *N. cyriacigeorgica*, *N. brasiliensis* and *N. nova* do not represent single species, but comprise three ANI species clusters each (Figure 1(Fig. 1)). The two strains from the German Collection of Microorganisms (DSMZ) sequenced in this study, namely, *N. lasii* (DSM 100525) and *N. wallacei* (DSM 45136), represent additional species, where no genome sequences have been available so far (Figure 1(Fig. 1)). Our patient isolates clustered across the entire tree spanning a wide phylogenetic range. In total, 13 different species were identified based on Average Nucleotide Identity (ANI) cut-off values of < 95 %. Two closely related strains isolated from two patients (P21_1 and P22_1) did not have any close references. The ANI of two further strains, P02_1 and P23_1, was also below 95 % with the closest relatives, i.e., *N. amamiensis* and *N. neocaledoniensis*, respectively (Figure 1(Fig. 1)). Thus, based on the genome phylogeny, those four strains can be considered as belonging to three novel *Nocardia* species.

Most *Nocardia* species, e.g. *N. farcinica* (n=8), *N. cyriacigeorgica* (n=6) and *N. veterana* (n=3), were cultured from respiratory specimens, whereas a few others, e.g., *N. brasiliensis* (n=2), were only cultured from non-respiratory specimens. Strains P22_1 and P23_1 belonging to an unknown species were isolated from a cystic fibrosis patient, who developed a pleura empyema one month after lung transplantation, and from an intraabdominal specimen from a different patient. A detailed summary of all strains with their site of infection can be found in Supplementary Table 1.

### Species identification of Nocardia strains based on low-cost, high-throughput techniques

We assessed the applicability of two MALDI-TOF protocols ((I), standard protocol with formic acid of bacterial colonies on the MALDI-TOF target plate and (II), improved protocol with formic acid and acetonitrile incubation of colonies before pipetting on the MALDI-TOF target plate) for species identification (Table 2(Tab. 2)). Most species, i.e. *N. farcinica* as the most common species, were identified to the species level using the standard (I) and the advanced protocol (II). Others, namely, *N. nova* and *N. wallacei*, were better identified using protocol (II). Overall, both protocols correctly identified 29 (74.4 %) and 35 (89.7 %) *Nocardia *strains to the species level, respectively. One strain (P02_1) belonging to a novel *Nocardia* species which is closely related to *N. amamiensis* was misidentified by both protocols as *N. abscessus*. Another novel *Nocardia* species (P23_1) was identified as the closest relative, *N. neocaledoniensis*. Two strains belonging to another novel species (P21_1 and P22_1) could not be identified via MALDI-TOF.

Results based on 16S rRNA gene analysis were mirroring those derived from WGS, where the same closest references to individual isolates were detected (Supplementary Figure 1). In accordance with WGS, 16S rRNA genes of strains P21_1 and P22_1 did cluster separately reflecting a novel species; in case of P02_1 and P23_1, sequences were closely related to those from *N. amamiensis* and *N. neocaledoniensis*, respectively.

### Antibiotic susceptibility testing

Results of microdilution-based antibiotic susceptibility testing of 37 patient strains are reported in Table 3(Tab. 3). Overall, we did not detect any linezolid (LZD) resistant strains. Except for *N. wallacei*, which has an intrinsically reduced amikacin (AMK) susceptibility, we did not detect any other AMK resistant strains. The overall SXT resistance rate was 13.5 %.

P21_1 and P22_1 that represent a novel *Nocardia *species did not grow in liquid culture media. One of these strains (P21_1) showed sufficient growth on solid culture media and we were, hence, able to perform E-test. The strain was tested susceptible for SXT (minimal inhibitory concentration (MIC) 2 mg/L), moxifloxacin (MXF) (MIC 0.25 mg/L), AMK (MIC 0.5 mg/L) and LZD (MIC 2 mg/L).

Furthermore, we compared microdilution-based susceptibility testing with MIC test strips for some therapeutically relevant antibiotics (SXT, MXF, AMK, and LZD). According to the CLSI recommendations, microdilution is the gold standard for antibiotic susceptibility testing; however, the broadly available MIC test strips could be an easy to implement method for antibiotic susceptibility testing. For both methods, we used *N. nova* ATCC BAA-2227 as the control strain. MIC values of the control strain were determined within the expected ranges with both methods. Twenty-six patient strains showed good growth on both solid culture media (for MIC strip tests) and liquid culture media (for microdilution). We did not detect any very major or major errors, while 8 (31 %), 4 (15 %), 1 (4 %) and 4 (15 %) minor errors for SXT, MXF, AMK and LZD, respectively, were recorded.

### Molecular analysis of antibiotic resistance

We used the CLC Genomics Workbench to BLAST the assembled *Nocardia* genomes against different resistance databases (CARD and QIAGEN Microbial Insights AR). Potentially relevant resistance mechanisms were detected in two *Nocardia* strains: (I) detection of dfrC (target replacement) in an *N. veterana* strain (P29_1), which leads to SXT resistance. This strain was also tested SXT resistant by microdilution. (II) Detection of dfrC and norA (efflux pump, which leads to chinolone resistance) in one *N. cyriacigeorgica* strain (P11_1). This strain was tested resistant against MOX, but sensitive against SXT. Other resistance mechanisms were not detected via this approach.

**Figure 2:**
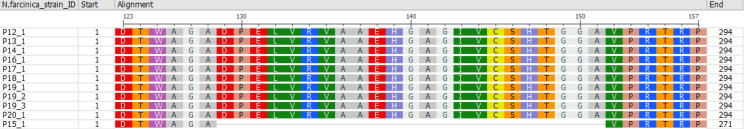
Multiple sequence alignment of the “inactive” FolP2 protein sequences from eleven *N. farcinica* isolates. Isolate P15_1 showed a phenotypic trimethoprim-sulfamethoxazole (SXT) resistance, whereas all other isolates depicted were SXT susceptible.

We looked into SXT resistance in more detail, as this is one of the most commonly used antibiotics in the treatment of nocardiosis. The SXT resistant *N. farcinica* isolate (P15_1) was compared with the remaining ten *N. farcinica* strains that were all SXT susceptible. Several enzymes are involved in the bacterial purine synthesis, which are inhibited by SXT. Dihydropteroate synthase (DHPS/ FolP) is inhibited by sulfamethoxazole, whereas dihydrofolate reductase (DHFR/ FolA) is blocked by trimethoprim. A third enzyme that might be crucial for purine synthesis was annotated as “inactive” homolog of FolP (FolP2) for all 11 strains. We did not find any mutations or deletions in SXT resistant and susceptible strains for FolP or FolA. However, isolate P15_1 showed a deletion of 23 amino acids (positions 129-151) in the “inactive” enzyme FolP2 that was absent in susceptible strains, most probably explaining resistance of this strain (Figure 2(Fig. 2)). 

## Discussion

Nocardiosis is a rare, but life-threatening disease and microbiological diagnostics are challenging. In our study, we described the epidemiology of nocardiosis in a tertiary care center in Germany with more than 140 lung transplantations per year. In contrast to data from the US (Woodworth et al., 2017[29]), we did not see an increasing incidence of nocardiosis in the study period from 2000 to 2018, which is in accordance with other reports from Germany and Western Europe (Ott et al., 2019[19]; Ercibengoa et al., 2020[11]). For robust subgroup analysis of disease incidence, the total patient number was too low in our study. 

There are incongruent results regarding the time point of diagnosis post transplantation. The median time between organ transplantation and first diagnosis of nocardiosis in our study was 13.5 month. Our findings are in contrast to Peleg et al. (2007[22]), who describe that the majority of SOT patients received the diagnosis of nocardiosis within one year after transplantation. However, also others (Santos et al., 2011[25]) described that the majority of SOT patients developed nocardiosis more than one year after transplantation, which is in line with our findings and highlight the importance of *Nocardia* specific diagnostics in this patient group over a long time post transplantation.

The distribution of *Nocardia* isolates was very broad and included many different species, suggesting that potentially any member of this genus can be considered as an (opportunistic) pathogen and the possibility of infection is not restricted to a few specific species. Interestingly, taxonomic affiliation differed between strains isolated from pulmonary and extra-pulmonary sites, in particular for *N. brasiliensis* that was only cultured from non-respiratory specimens of two immunocompetent patients. This observation is in line with case reports describing *N. brasiliensis* as a cause of non-pulmonary nocardiosis in immunocompetent patients (Zhu et al., 2020[31]; Chen et al., 2020[8]). Furthermore, we describe the first isolations of three novel* Nocardia* *spp.* from patient specimens, where specifically strains P21_1 and P22_1 lacked any close reference. Unfortunately, these strains did not grow in Mueller-Hinton broth with TES and therefore we were unable to generate microdilution-based antibiotic susceptibility results. However, one strain (P21_1) showed sufficient growth on solid culture media and we were able to generate MIC strip test-based results. These results showed that the most commonly used antibiotics for the treatment of nocardiosis, SXT, LZD or MXF, were active on this strain.

Both high-throughput identification methods tested here, MALDI-TOF and 16S rRNA gene analysis, were evaluated as effective for the identification of *Nocardia* strains and showed good accordance with WGS results. Data derived from MALDI-TOF considerably improved using the advanced protocol established with formic acid and acetonitrile. This is in line with the findings of Marín et al., who described MALDI-TOF as a good tool for species identification of *Nocardia* (Marín et al., 2018[15]). They applied a similar MALDI-TOF protocol using, however, another MALDI-TOF manufacturer (Bruker) and a different database. Additionally, Body et al., used the Vitek-MS MALDI-TOF system with the IVD 3.0 database (Body et al., 2018[6]). They identified 236 (76 %) of their *Nocardia* strains (n=312) down to species level and an additional 44 (14 %) strains at the complex level. With up to 90 % correct species identification rates, the Saramis database used in our study revealed better identification results, however, it represents an open research use only database and results have to be interpreted with caution. 16S rRNA gene analysis results were mirroring those derived from WGS, indicating that 16S rRNA-based identification is a useful tool for *Nocardia* species identification. Both methods are broadly available in diagnostic microbiology laboratories and cost effective, making more advanced molecular methods like MLST or WGS not essential for correct strain identification in a routine diagnostic laboratory. However, for rare species or, as shown here, for novel species that are not included in the MALDI-TOF database or in the 16S rRNA reference database molecular analysis based on WGS is helpful for correct species identification. Two closely related isolates (P21_1 and P22_1) were not phylogenetically close to any publicly available reference genome, and an additional two strains*,* P02_1 and P23_1, displayed ANI values of > 93 % and < 95 % to the closest references, which is below the currently accepted ANI species cut-off of 95 % (Chun et al., 2018[9]). However, values in particular of the latter two were close to this cut-off, and 16S rRNA gene analysis classified them as their closest references, while MALDI-TOF misidentified strain P02_1. Additional analyses, including biochemical testing, are needed to decide whether they truly belong to novel species.

Currently, it is controversially discussed whether antibiotic susceptibility testing via MIC strip tests is a reliable technique in comparison to the gold standard microdilution to determine antibiotic resistance of *Nocardia* *spp.* (Biehle et al., 1994[5]; Ambaye et al., 1997[1]). In our study, 26 different *Nocardia* strains from patient specimens were available for comparative testing via both methods. We did not find major or very major errors comparing these two methods. However, we found minor errors with MIC discrepancies of more than one titration step for SXT, MXF and LZD. These discrepancies did not lead to a differing categorization as susceptible, intermediate or resistant. Thus, MIC strip tests might be a suitable alternative for broth microdilution-based testing, at least when microdilution techniques are not applicable.

Molecular mechanisms of antibiotic resistances for *Nocardia* are poorly understood and resistance databases such as CARD do, currently, not include specific resistance mechanisms of *Nocardia* (McArthur et al., 2013[16]). Therefore, it is not surprising that these databases were not suitable for molecular antibiotic resistance detection in our study. We looked further into possible mechanisms of SXT resistance, as this is one of the first line antibiotics for the treatment of nocardiosis. In a previous study, Valdezate et al. (2015[28]) analyzed different *Nocardia spp*. exhibiting SXT resistance by targeted amplification of genes that are involved in the folate biosynthesis pathway. They found mutation in both dihydrofolate reductase (DHFR) and dihydropteroate synthase (DHPS) causing SXT resistance; however, they suggested that there might be additional resistance mechanisms. In a recent study, Mehta et al. (2018[17]) described different mechanisms of SXT resistance for *N. cyriacigeorgica* and *N. nova*. They adapted these strains to SXT to analyze the evolution of SXT resistance. The observed resistance mechanisms were based on mutations and/or deletions in three enzymes that are involved in the bacterial purine synthesis, namely, FolA, FolP and “inactive” FolP2. Based on these observations, we analyzed another common species, *N. farcinica,* and identified a deletion of several amino acids in FolP2 in the resistant strain, while the other two enzymes FolA and FolP were unaffected on the sequence level. The resistance mechanism was detected in a clinical patient derived strain demonstrating that this mechanism may also play an important role *in vivo, *and involves distinct* Nocardia *species*.* Our observation underlines previous findings (Mehta et al., 2018[17]) that besides FolA and FolP, FolP2 seems to be crucial for sulfamethoxazole mechanism of action and that FolP2 does not seem to be inactive (as annotated by prokka) in wild-type strains. The sequence-based approach applied here for inferring antibiotic resistance demonstrates the potential for gathering functional, clinically relevant data at the DNA level. Metagenomic analyses of samples obtained from patients might, hence, be a powerful tool for not only assigning species names, but additionally predicting their antibiotic resistance *in silico*, thereby circumventing the need for the tedious cultivation of respective bacteria.

## Notes

Patrick Chhatwal and Marius Vital (Institute for Medical Microbiology and Hospital Epidemiology, Hannover Medical School (MHH), Carl-Neuberg-Str. 1, Gebäude J6, 30625 Hannover, Germany; E-mail: vital.marius@mh-hannover.de) contributed equally as corresponding author.

## Declarations

### Ethics approval

We obtained ethical approval for this study from the ethics committee of the Hannover Medical School (No. 8504_BO_K_2019).

### Data availability

All data generated or analyzed during the current study are included in this published article. Sequence data generated during this study are available from the European Nucleotide Archive (https://www.ebi.ac.uk/ena/) under the accession number PRJEB43439.

### Competing interests

The authors declare that they have no competing interests.

### Funding

This research did not receive any specific grant from funding agencies in the public, commercial, or not-for-profit sectors.

### Authors' contributions

All authors contributed to the manuscript according to the ICMJE (International Committee of Medical Journal Editors) recommendations: PC, MV and DS: preparation of the manuscript. PC organized the drafting process. PC, DS, MV, TW: design and concept of the study. SW, PC, MV and SZ: data acquisition, performance of experiments, data analysis. All authors critically revised the manuscript, account for accuracy and correctness and have read and agreed to the final draft before submission.

### Acknowledgments

We thank Tanja Katzorke for performing the MALDI-TOF analysis and the whole team of the Institute for Medical Microbiology and Hospital Epidemiology of the Medical School Hannover for the isolation and the storage of the *Nocardia* *spp.* from patient specimens.

## Supplementary Material

Supplementary information

## Figures and Tables

**Table 1:**
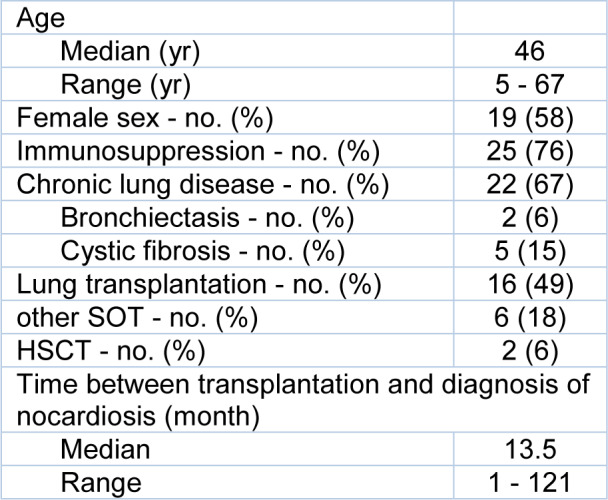
Baseline characteristics of Nocardiosis patients (n=33). SOT = solid organ transplantation; HSCT = hematologic stem cell transplantation

**Table 2:**
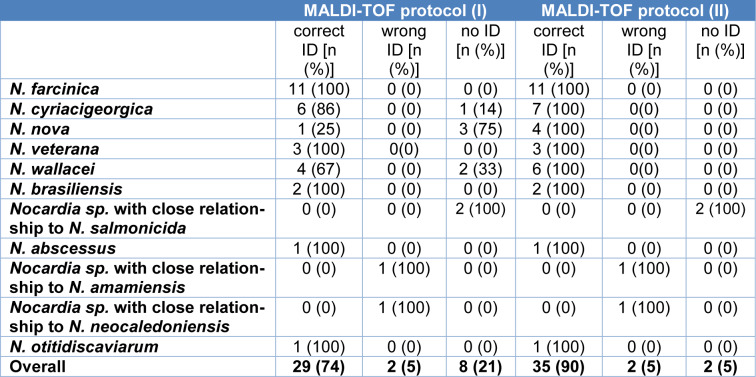
Comparison of MALDI-TOF-based identification protocols. The reference method was whole genome-based ANI values. No ID is defined as no identification to the species level.

**Table 3:**
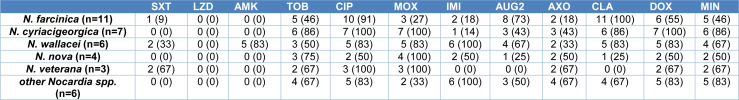
Antibiotic susceptibility testing results of patient strains based on microdilution (n=37). Resistance rates: n (resistant) (%)
