# Supplementary material for: Molecular characterization and improved diagnostics of Nocardia strains isolated over the last two decades at a German tertiary care center
Source: EXCLI J. 2021 Apr 30;20:851–62. doi: 10.17179/excli2021-3787 (PMC8222631; doi:10.17179/excli2021-3787)
Supplement: Supplementary information [file EXCLI-20-851-s-001.pdf]

EXCLI Journal 2021;20:851-862 – ISSN 1611-2156
Received: April 18, 2021, accepted: April 22, 2021, published: April 30, 2021

Supplementary information to:

Original article:

MOLECULAR CHARACTERIZATION AND IMPROVED DIAGNOSTICS OF *NOCARDIA* STRAINS ISOLATED OVER THE LAST TWO DECADES AT A GERMAN TERTIARY CARE CENTER

Patrick Chhatwal[1*], Sabrina Woltemate[1], Stefan Ziesing[1], Tobias Welte[2], Dirk Schlüter[1], Marius Vital[1*]

[1] Institute for Medical Microbiology and Hospital Epidemiology, Hannover Medical School (MHH), Hannover, Germany
[2] Department of Pneumology and German Center for Lung Research, Hannover Medical School (MHH), Hannover, Germany

* **Corresponding authors:** Marius Vital and Patrick Chhatwal, Institute for Medical Microbiology and Hospital Epidemiology, Hannover Medical School (MHH), Carl-Neuberg-Str. 1, Gebäude J6, 30625 Hannover, Germany.
E-mails: vital.marius@mh-hannover.de; chhatwal.patrick@mh-hannover.de

http://dx.doi.org/10.17179/excli2021-3787

This is an Open Access article distributed under the terms of the Creative Commons Attribution License (http://creativecommons.org/licenses/by/4.0/).

EXCLI Journal 2021;20:851-862 – ISSN 1611-2156
Received: April 18, 2021, accepted: April 22, 2021, published: April 30, 2021

Supplementary Table 1: Overview of all *Nocardia* strains with their site of infection

Strain	WGS-based identification (ANI to the closest neighbor [%])	Site of infection	Immunosuppression
**P01_1**	*Nocardia abscessus* NBRC 100374 (95.6)	respiratory tract	no
**P02_1**	*Nocardia amamiensis* NBRC 102102 (93.9)	respiratory tract	no
**P03_1**	*Nocardia brasiliensis* IFM 10847 (98.7)	elbow bursitis	no
**P04_1**	*Nocardia brasiliensis* IFM 10847 (98.7)	facial cellulitis and lymphadenitis	no
**P05_1**	*Nocardia cyriacigeorgica* (98.3)	respiratory tract	yes
**P06_1**	*Nocardia cyriacigeorgica* (99.7)	respiratory tract	yes
**P07_1**	*Nocardia cyriacigeorgica* GUH-2 (98.4)	respiratory tract	no
**P08_1**	*Nocardia cyriacigeorgica* GUH-2 (98.2)	respiratory tract	yes
**P09_1**	*Nocardia cyriacigeorgica* (97)	respiratory tract	yes
**P10_1**	*Nocardia cyriacigeorgica* GUH-2 (98.3)	brain abscess	yes
**P11_1**	*Nocardia cyriacigeorgica* GUH-2 (98.3)	respiratory tract	yes
**P12_1**	*Nocardia farcinica* (99.4)	respiratory tract	yes
**P13_1**	*Nocardia farcinica* (99.3)	pleural infection	yes
**P14_1**	*Nocardia farcinica* (99.1)	respiratory tract	no
**P15_1**	*Nocardia farcinica* (99.3)	respiratory tract	no
**P16_1**	*Nocardia farcinica* (99.1)	respiratory tract and brain abscess	yes
**P17_1**	*Nocardia farcinica* (99.1)	respiratory tract	yes
**P18_1**	*Nocardia farcinica* (99.2)	respiratory tract	yes
**P19_1**	*Nocardia farcinica* (99.1)	respiratory tract	yes
**P19_2**	*Nocardia farcinica* (99)	respiratory tract	yes
**P19_3**	*Nocardia farcinica* (99.1)	respiratory tract	yes
**P20_1**	*Nocardia farcinica* (99.1)	respiratory tract and throat abscess	yes
**P21_1**	*Nocardia salmonicida* NBRC 13393 (89)	pleural infection	yes
**P22_1**	*Nocardia salmonicida* NBRC 13393 (89)	intraabdominal infection	no
**P23_1**	*Nocardia neocaledoniensis* (93.2)	respiratory tract	yes
**P24_1**	*Nocardia nova* (99.3)	tenosynovitis hand	yes
**P25_1**	*Nocardia nova* NBRC 15556 (99.5)	respiratory tract	yes
**P26_1**	*Nocardia nova* (99.2)	respiratory tract	yes
**P26_2**	*Nocardia nova* (99.2)	respiratory tract	yes
**P27_1**	*Nocardia otitidiscaviarum* IFM 11049 (98.1)	central nervous system	yes
**P28_1**	*Nocardia veterana* NBRC 100344 (98.9)	respiratory tract	yes
**P29_1**	*Nocardia veterana* NBRC 100344 (98.9)	respiratory tract	yes
**P30_1**	*Nocardia veterana* NBRC 100344 (98.9)	respiratory tract	yes
**P31_1**	*Nocardia wallacei* (99.3)	respiratory tract	yes
**P32_2**	*Nocardia wallacei* (99.4)	respiratory tract	yes
**P32_3**	*Nocardia wallacei* (99.3)	respiratory tract	yes
**P32_4**	*Nocardia wallacei* (99.4)	respiratory tract	yes
**P32_5**	*Nocardia wallacei* (99.4)	respiratory tract	yes
**P33_1**	*Nocardia wallacei* (99.1)	respiratory tract	yes

EXCLI Journal 2021;20:851-862 – ISSN 1611-2156
Received: April 18, 2021, accepted: April 22, 2021, published: April 30, 2021

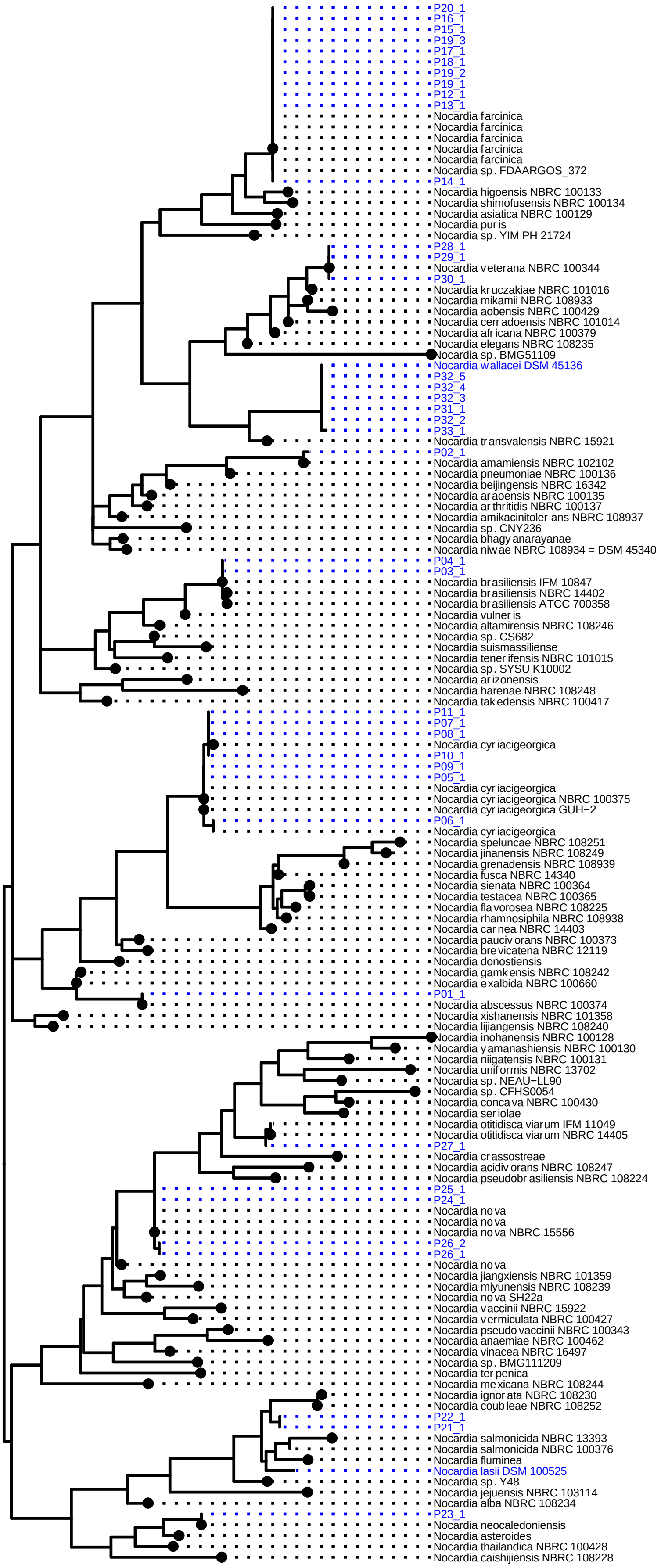

Supplementary Figure 1: Phylogenetic tree based on 16S rRNA genes from all isolates of this study (blue) and from *Nocardia* reference genomes of the Genome Taxonomy Database (GTDB), where all closest matching references (based on Average Nucleotide Identity shown in Figure 1) as well as all representative genomes of individual species (highlighted with a dot at the tip) were included. The two references from the German Collection of Microorganisms (DSMZ) sequenced in this study are shown in blue.
